# European Union Child Guarantee—challenges raised by the welcome promise of free healthcare for marginalized children

**DOI:** 10.1093/eurpub/ckab062

**Published:** 2021-05-29

**Authors:** Michael J Rigby

**Affiliations:** 1 School of Social, Political and Global Studies, Keele University, Keele ST5 5BG, UK; 2 School of Primary, Community and Social Care, Keele University, Keele ST5 5BG, UK

## Abstract

**Background:**

Children are dependent on the way in which society provides healthcare, with primary and preventive care being initial components. They also have a generally acclaimed right to health, and to lack of impediment to access to healthcare. In a major initiative, the European Parliament has proposed a Child Guarantee to include free access to healthcare for marginalized children, and a Feasibility Study has been completed with positive results. However, there has been little analysis of national policies toward free access to healthcare for children, including longer-term treatment, mental health or adolescent health services, or of charges and indirect financial barriers to access

**Methods:**

Data on policies for children’s access to healthcare from two recent European Community wide studies were re-analyzed and matched. Primary care, immunization, surveillance screening, minor illness, a more significant medium-term condition, and reproductive health were included. Additionally, data from a European survey of children reported as having unmet medical needs were revisited. Composite summaries relating to all 28 EU countries as of 2019 were produced.

**Results:**

Only three EU-28 countries provided totally free services, though 26 countries provide free primary and preventive services. There is evidence of some children having unmet medical needs in 21 countries, with Expense being the main quoted factor.

**Conclusion:**

There is widespread variation across Europe in free access for children to healthcare, little comparative study of policies and their effects on enabling or hindering access, and minimal data collection. This compromises achievement of the Guarantee, and initiatives are needed.

## Introduction

Within the European Union (EU), healthcare is a national competence and each country controls its own health policy. Eurostat produces comparative statistics, and the Commission itself undertakes focussed inter-state activities in specific fields such as reference networks for rare diseases, and aspects of public health planning.

The Commission has facilitated a major initiative in the health and healthcare of the elderly, focussed on health rather than just health systems.^1^ In contrast, comparatively little attention has been paid to healthcare for children, other than some single topic actions such as in support of immunization. However, there is known to be considerable variety in children’s healthcare provision across the EU, including differences in funding models, delivery structures, and professional roles.^2–5^

### A new recognition of children including their health

All EU member states are signatories to the United Nations Convention on the Rights of the Child (UNCRC),^6^ and there is active monitoring by the EU of member states’ compliance. More recently, EU bodies have realized that children are important as the forthcoming adult population as well as citizens in their own right. In 2013, the Commission issued a Recommendation on Investing in Children—Breaking the Cycle of Deprivation^7^ which emphasized the importance of investing in infrastructure and services in order to reduce economic and other circumstances adversely affecting children—though this guidance was holistic and not specific to health. Recognizing the importance of vaccination and concerned about declining rates, the Council of Europe produced initiatives, though outside the context of overall integrated child health preventive programmes.^8^^,^^9^ Most recently, the EU Expert Panel on Effective Ways of Investing in Health has considered immunization, including looking at barriers to uptake.^10^

### Proposed EU ‘Child Guarantee’

The new significant development in supporting marginalized children came from the European Parliament, which in 2017 asked the Commission to examine the concept of a European Child Guarantee for marginalized children of ‘free healthcare, free education, free early childhood education and care, decent housing and adequate nutrition’.^11^^,^^12^ This makes the promise of a free healthcare service for marginalized children central, but without defining either ‘healthcare’ or ‘free’. This impedes intentions to fulfil the Guarantee, as healthcare and financial barriers are complex and inadequately understood and therefore guaranteeing them is compromised.

In order to introduce factual evidence to the discussion, this article uses data from two recent EU-wide studies, and other material including survey statistics, to assess the situation regarding free child healthcare policy in EU member states. It also highlights the minimal data on healthcare need or delivery to children. Addressing these knowledge gaps will be essential to progressing the healthcare promise which leads the Child Guarantee.

## Methods

Two recent EU-wide studies, key reports, and recent databases were analyzed to highlight the current inadequate knowledge of ‘free’ children’s healthcare, financial barriers, and unmet need. The Models of Child Health Appraised (MOCHA) project assessed primary healthcare systems for children in the then 28 EU Member States and two European Economic Area countries.^13^^,^^14^ The project retained a national expert in each country,^15^^,16^ and considered aspects of service access including charges. Shortly afterwards the EU-commissioned Feasibility Study of the Child Guarantee^12^ was undertaken by a non-governmental consortium which commissioned a children’s services expert in each country to obtain policy data for the five service areas of the proposed Guarantee; while not necessarily a health expert, at minimum they could give an informed lay view of child health provision.

This article also links results on charges for children’s health services from these two studies with other evidence of economic barriers to children accessing healthcare. This identifies essential prerequisites to enabling progress with the ‘free healthcare’ aspect of the Child Guarantee.

## Results

### Access costs for children’s primary care

**Figure 1:**
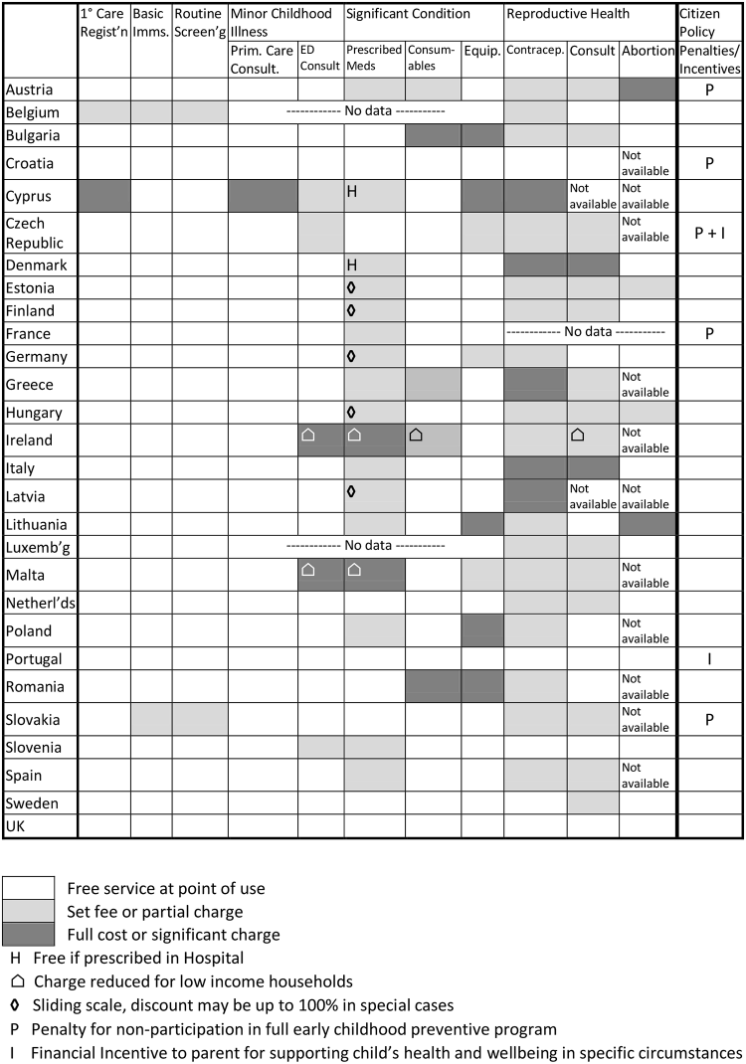
Principles of charging in EU member states for Eleven child health services

The meta-analyses of aspects of health service provision for children are discussed sequentially, and summarized in figure 1.

The first topic was whether basic primary care for children is free. The MOCHA study found that 25 of 26 countries had no registration or enrolment charge. In Cyprus, with an insurance-funded health system, each insurance company charges an insurance premium.

Basic childhood immunization was studied as a core service, and data obtained for 25 countries. Though each country has its own immunization schedule, all had a free core service. In Italy, Latvia, Lithuania and the Netherlands the publicly funded free service was for the main childhood immunizations only; in Poland the free service was for provision by single antigen while there was a charge for seeking a combined vaccine.

Screening and routine examinations are also a key part of children’s preventive healthcare, and the MOCHA project obtained policy data for 25 EU Member States—all had defined screening programmes. Though the countries’ schedules were very different, none of them charged a fee (though some had a private fee-based option as an alternative to the free public service).

However, primary care is clearly not restricted to planned preventive care. Childhood illness happens, and except for more serious accidents primary care should be the first source of advice and treatment. However, every health situation is a different personal story. The MOCHA study therefore assessed policy and pattern of provision, charging, and funding in each EU country for several scenarios.

First was short-term illness of a well child, with assessment of whether there was a cost for consultation, and for prescribed pharmaceuticals. The scenario postulated was of a 2-year-old child quickly developing a mild fever, and rash, on a weekday afternoon, being clearly uncomfortable; the parents decide they want their child to be seen by a health professional within 24 hours. Service responses were obtained for 25 countries—all countries would be able to offer an urgent appointment with the primary care provider, and except in Cyprus there would be no fee. All countries except the Netherlands could offer either an urgent referral centre appointment or a hospital Emergency Department service, and there was no charge for the hospital service except in Cyprus, Czech Republic, Slovakia, and Ireland. In the first three of these the charge was modest; in Ireland there was no charge if the child was already registered for a Medical Card as a result of chronic illness or low family income. Thus in all countries a primary care response was not charged, and urgent attendance hospital costs were absent or minimal except for healthy children in Ireland not from poor families.

### Prescribing charges

However, countries’ systems were not all as financially amenable for dispensing costs for pharmaceuticals prescribed for the child. Table 1 shows the situation with prescribing fees in 25 EU countries. Only nine countries (36%) supply free medication for primary care consultations (and in the case of Portugal this is only for children under 12 years); in most of these countries there is a national list of approved pharmaceuticals for free or nominal fee dispensing. Three more countries reimburse on a sliding scale according to the pharmaceutical product—this may be up to 100% reimbursement, while Latvia discounts according to diagnosis. Two more countries provide free dispensing in hospital, but not for primary care. Only Malta and Ireland have no fee, or a nominal fee, specifically for poorer families; only Hungary and Ireland have no or a nominal fee for children with a chronic condition. In other countries there is either a nominal or percentage payment, but with only Malta and Ireland having the full cost as the normal charge unless the family has a low income. Thus for most children in Europe there is no free medication provision for minor childhood illness, though for most there is a subsidy.

**Table 1:** Prescribing charges—generally well-child with childhood fever

	No fee	No fee if low income	No fee if chronic illness	Small flat fee	Full cost	Other
Austria				√		
Belgium	No data					
Bulgaria	√^a^					
Croatia	√					
Cyprus	√ H					Private: General Practitice full cost (reimbursed if insured); Emergency Department: fee covers also drugs
Czech Republic	√^a^					
Denmark	√ H					General Practice: co-payment; Hospital: free
Estonia						Pharmacy price discounted by up to 100%
Finland						Pharmacy price discounted by up to 100%—€4.50 fee
France						Partial reimbursement
Germany						Sliding scale according to pharmaceutical
Greece						25% of cost + €1
Hungary			√			Sliding scale according to pharmaceutical; free for children with listed chronic disease
Ireland		√^b^	√^b^	√^b^		Non-Medical Card children—full cost up to annual ceiling
Italy				√		
Latvia						Pharmacy price discounted by up to 100% depending on diagnosis
Lithuania						Depending on drug
Luxembourg	No data					
Malta		√			√	
Netherlands	√^a^					
Poland						Free or discounted depending on product
Portugal	√ under 12 years					Partial reimbursement if prescribed
Romania	√^a^					
Slovakia	√^a^					
Slovenia	No data					
Spain						Proportional co-payment
Sweden	√					
UK	√					

Note: H, Hospital prescription only.

Source: MOCHA Project.^13^

a If on national list of approved fully fundable (or partly fundable) pharmaceuticals.

b Low income or child with chronic disease—Medical Card means government picks up cost less €2.50.

### Consumables charges in children’s healthcare

A second case represented more complex conditions with sudden onset and ongoing healthcare costs. The scenario was a baby born with a cleft palate, but otherwise healthy, requiring naso-gastric feeding, and airways suctioning through an in-dwelling tube. After three weeks in hospital, she is well enough to be discharged home for six months, pending surgical correction. The question posed concerned payment for the consumables necessary for naso-gastric feeding, and the pump needed to suction her airways.

Table 2 shows that in 16 of 25 reporting countries the consumables are provided free; in 9 the parents meet a full or partial cost. Regarding the suction pump, in 16 countries this is provided free, leaving 9 countries where the parents make some financial contribution—arrangements are often local, discretionary and complex. In summary, in one-third of countries this is not free, and parents face sudden health costs, usually partial costs but in some countries full costs. In a few countries these charges could be catastrophic, and are an added trauma on top of the sudden health condition.

**Table 2:** Charges for consumables and equipment—infant with cleft palate living at home

	Funding of consumables such as disposable tubes, feeding syringes and testing strips								Funding of short-term equipment such as portable suction pump				
	Hospital supplies for free	Community or primary health services provide for free	Community pharmacy dispenses for free	Health insurance reimburses within basic cover	Health insurance reimburses only if the parents/child have a higher level of insurance.	Parents purchase at full cost	Parents contribute some of the cost (co-payment)	Loaned for free by the hospital	Loaned for free from community or primary health services	Community pharmacy dispenses the pump for free during the time it is needed	Health insurance reimburses within basic cover	Parents purchase at full cost	Parents contribute some of the cost (co-payment)
Austria				X			X	X		X^a^			
Belgium	No data												
Bulgaria						X^b^						X^c^	
Croatia	X	X	X							X			
Cyprus	X											X	
Czech Republic				X			X						X
Denmark		X							X				
Estonia	X^d^						X^b^	X					
Finland		X							X				
France	X							X					
Germany				X			X				X		X
Greece							X						
Hungary			X							X			
Ireland		X^e^					X^b^		X				
Italy	X								X				
Latvia		X			X			X					
Lithuania				X			X					X	
Luxembourg	No data												
Malta	X												X
Netherlands				X							X		
Poland	X	X										X	X^f^
Portugal	X	X							X				
Romania						X						X	
Slovakia				X							X		
Slovenia	No data												
Spain		X							X				
Sweden		X								X			
UK		X						X	X				

Source: MOCHA Project.^13^

a Medical store supplies it.

b Some *ad hoc* support.

c Some *ad hoc* support.

d Retrospective reimbursement.

e If family has Medical Card for low income or chronic disease (if apply at this point there is a processing period and no retrospective reimbursement); otherwise, ceiling of €144 per month, tax allowable.

f Partial reimbursement if child can be registered as disabled.

### Charges for reproductive health services for older children

The MOCHA study also examined charges for an important issue for older children—reproductive health. For children starting to act autonomously, financial barriers to service access may have significantly adverse consequences. Table 3 shows the findings for 27 countries. Eleven countries make condoms available free of charge, and a 12th in a targeted way related mainly to HIV prevention. Another ten countries felt that widespread access through general retail outlets was an accessible and affordable service. In contrast, oral contraceptives were only available free of charge in nine countries, and at a token charge in another two. On reproductive health services, particularly availability of ‘morning after’ emergency contraception, table 3 shows that only seven countries provide a free service, in one it is covered by health insurance, in two countries the charge is nominal, and some charge only for the contraceptive. Poland reports that though the service is free, delays in getting an appointment may necessitate seeking a full-cost private appointment.

**Table 3:** Charges for adolescent reproductive health services

	Are free or low cost condoms available to adolescents?	Free oral contraceptives available to adolescents?	Are there charges for young persons’ emergency Reproductive Health Services?	Is there a fee for abortion?
Austria	Yes	No	Pay for emergency contraceptive	Yes
Belgium	Yes	No	Insurance covers	Insurance/national funding
Bulgaria	Yes	No	Pay for contraception	Insurance/national funding
Croatia	Yes	Yes	Free	NA
Cyprus	No	No	NA	NA
Czech Republic	Yes	No	Pay for emergency contraceptive	NA
Denmark	No	No	Pay in full	Insurance/national funding
Estonia	Retail	No	Retail	Co-payment
Finland	Retail	Yes	Retail	Insurance/national funding
France	No data			
Germany	Retail	Yes	Free	Insurance/national funding
Greece	No	No	Prescription	NA
Hungary	Retail	No	Prescription	Yes
Ireland	Retail	No	^a^	NA
Italy	No	No	Pay	Insurance/national funding
Latvia	No	No	NA	NA
Lithuania	Retail	No	Free	Yes
Luxembourg	Yes	Token charge	Token charge	Insurance/national funding
Malta	Yes	No	Pay for emergency contraceptive	NA
Netherlands	Retail	Yes	Pay for emergency contraceptive	Insurance/national funding
Poland	Retail	No	Free (but may need private for speed)	NA
Portugal	Yes	Yes	Free	Insurance/national funding
Romania	Retail	Yes	Free	NA
Slovakia	Retail	No	Pay for emergency contraceptive	NA
Slovenia	Yes	Yes	Free	Insurance/national funding
Spain	Targeted	Token charge	Token charge	NA
Sweden	Yes	Yes	Pay for emergency contraceptive	Insurance/national funding
UK	Yes	Yes	Free	Insurance/national funding

Source: MOCHA Project.^13^

a If family has Medical Card for low income or chronic disease (no retrospective reimbursement); otherwise, ceiling of €144 per month, tax allowable.

Finally, fees and charges for a termination of unplanned pregnancy vary, and in eleven countries this provision is not available. In twelve countries this procedure was covered by the normal insurance or national service funding without a fee. One country had a co-payment, and three charged up to full cost.

### Feasibility study validation of MOCHA study data

The MOCHA data were obtained by structured enquiries to a child health expert in each country. The later Feasibility Study for a Child Guarantee (FSCG) used generic children’s services experts to give a largely narrative report of children’s access to health services. The country and healthcare narrative reports are unpublished working documents, but a high level summary and the list of informants appear in the Intermediate Report.^12^ These FSCG narratives have no discordance with the MOCHA data, and enabled completion of data gaps, enabling the composite summary in Figure 1.

### Penalties and incentives targeted at parents

Some countries have policies which seek to influence parents towards ensuring that children attend preventive health programmes, effectively giving a cash cost to not availing of the free services. Those identified are:

- Austria—child benefit reduced if the parent cannot provide a fully completed MutterKindPass (the Austrian parent-held record^17^).
- Croatia—rules exist for reduction in welfare payments if there is non-compliance with the immunization schedule (though this is seldom applied).
- Czech Republic—authority to impose fines for non-compliance with the immunization schedule (though this is seldom applied).
- France—authority for child welfare cuts, and for criminal fines, for non-compliance with immunization.
- Slovakia—potential for financial penalties for non-immunization (poor families exempted); also for halving of welfare payments if parents do not follow the preventive health programme

Meanwhile, two countries have financial Incentives policies:

- Czech Republic—financial incentives for immunizations outside core free service, and for participation in specific health and mental health lifestyle programmes prescribed for those with particular needs
- Portugal—grant system for one parent to look after a child’s illness or accident-based needs if both parents work.

### Summary of free access and charging policies

The MOCHA project and the Feasibility Study for the Child Guarantee produced congruent results, and these free provision and charging policies are summed up in Figure 1. Only Croatia, Portugal and the UK have totally free services. All countries except Belgium and Cyprus provide free primary care, and all countries provide free basic immunization and routine screening, though Belgium and Slovakia limit financial coverage. Ireland and Malta have fee waivers for economically marginalized children.

For minor illness most countries enable free consultation, but only nine have totally free medicines. For a significant sudden onset condition such as a neonate with a cleft palate, this would present parents with some degree of financial burden in nearly all countries. In contrast, for an unplanned teenage pregnancy in 16 countries where a termination could be available there is no charge in 12, though only 10 countries make oral contraceptives available without charge.

### Other costs and financial barriers

However, charges for services are not the only costs incurred by parents in availing of children’s healthcare, and two recent papers looking at childhood immunization have highlighted these.^10^^,^^18^

Out-of-pocket costs which may be incurred include:

- Transport: fares or fuel, and parking fees. Moreover, working parents may have to travel from work to the child’s school or day-care location, then to the medical appointment, then back. Parking charges may also be incurred.
- Loss of earnings: in many marginalized families both parents, and single parents, are in employment, and this may be a social welfare requirement. To take a child for even a 5-minute appointment will require longer time off work; employers may require a full half-day to be forfeited.
- Extra childcare: if the appointment time means that the parent is not able to collect other children from school or pre-school at the normal time, there may be a further child minder or after-school fee.
- Food and incidentals: particularly for longer appointments or travel, there may be necessity to purchase food.

These are not health system fees, but they are unavoidable out-of-pocket costs of ensuring a child receives healthcare. Families with insecure housing or in emergency accommodation may be housed away from their normal locality, and have longer journeys and higher costs to keep continuity of healthcare service. Families in precarious situations are also most likely to be in employments with least flexibility for short absences.

In summary, even where there is a ‘free’ service, there may be considerable parental expense to obtain it. Paradoxically, marginalized families may face the highest out-of-pocket costs.

### How important is a free service for children?

It may seem that the need for free health services for children is unnecessary, but this lack of understanding is due to the unacceptably limited data about children’s health needs and services.^19^ The one European data source on unmet needs and financial barriers, perforce used by the FSCG, is an *ad hoc* module in 2017 within the Eurostat Statistics on Income and Living Conditions (SILC).^20^ The approach is flawed, but even so results indicate that possibly 1 million EU children have unmet medical needs; in seven countries this comprised 2% or more of children.^21^

Reported reasons are presented for 12 countries; in seven ‘Expense’ is the biggest reason; in three countries ‘Too Far’ and ‘No Time’ are together important causes. Belgium and Cyprus, the two countries without free primary care, have significant economic barriers to children receiving treatment. Thus, though imperfect, the SILC data confirm financial barriers to access.

## Discussion

‘Free healthcare’ is the lead promise of the Child Guarantee. This meta-analysis shows that it only exists in two EU countries post-Brexit, though Malta and Ireland have cost waivers for economically marginalized children. Thus considerable work is needed within the health sector, and the health services of Member States, to enable progression of this concept and its achievement. Disappointingly, neither the Health nor Research Directorates of the European Commission were included in the sponsorship of the Feasibility Study or its follow-on, underscoring the need for health sector stimulus of action.

### What is realistic free healthcare provision?

A key objective of the World Health Organization (WHO) is Universal Health Coverage, whereby ‘all people have access to the health services they need, when and where they need them, without financial hardship’.^22^ Meanwhile, the European Regional Office of WHO has consistently emphasized a strategic approach to child health.^23^^,^^24^ The UNCRC specifies: ‘States Parties shall strive to ensure that no child is deprived of his or her right of access to such healthcare services’.^5^ Thus the free healthcare aspect of the Child Guarantee could be considered overdue, and rather than being innovative is in fact doing no more than promoting established basic principles. But, what has been shown is not only the lack of children’s unimpeded healthcare access, but lack of measures, knowledge or means.

The Feasibility Study indicated the range of services necessary to achieve ‘healthcare’, including mental health, dental health, and stronger prevention services including health literacy. To be able to assess effective free access it is necessary to test against a representative package of example core services—this was hypothesized during the Feasibility Study and the respondents for Cyprus, Hungary and Slovenia found it workable as a reporting frame.^12^ A concerted move to improve data on child health in Europe, including frameworks for defining necessary healthcare and free access for marginalized children (and lack of barriers for any children), is urgently needed.^12^^,^^19^^,^^21^^,^^25^

### Improving knowledge

As well as data improvement, knowledge of better service delivery methods, including identification of marginalized and other at risk children, and how to get services to them without economic (or organizational) barriers, is crucial. There is much scope for evidence exchange, and supported research. The analyzed studies underscore how limited is knowledge of children’s access to healthcare, and the economic and practical barriers experienced by marginalized children. Even facts on why children do not keep appointments are not systematically recorded though this could be key to facilitating access.^26^ Individual FSCG country representatives also indicated how each country could improve accessible free services for marginalized children, summated in an Appendix (Annex 7.5: Main priorities to improve access to effective and comprehensive free healthcare).^12^ More recently some national initiatives are progressive, such as.^27^

## Conclusion

The Child Guarantee makes a bold promise about marginalized children’s access to free healthcare. Analysis indicates that this is much overdue according to established polices, yet most countries in Europe need to take to action to ensure that all children have practical universal access to healthcare without financial or other barriers. Children in low income, homeless and other marginalized families are most at risk. Only two EU-27 countries promise a free healthcare service, and another two report systems to address economic barriers for low-income families or children with chronic conditions. However, the issues are not just of healthcare provision, and of ensuring there are no direct health system costs, but also that other supports are available including social welfare support to cover travel and other indirect costs.

When young, children need to have access to preventive and primary healthcare without their parents being put off by direct or out-of-pocket expenses. Resolving some of the latter is likely to require cross-sectoral collaboration, including social welfare funding; also timing and location of service access. Additionally, not all countries shield parents from the economic shock of sudden ongoing healthcare costs for a more serious condition.

As children grow older and start to act autonomously, free access to services such as mental health and reproductive health are very important,^23^ but the individual child is unlikely to have personal means to pay. Involving parents in payment would in many cases result in failure to access—the adverse outcomes of this can include self-harm and suicide, unsafe sexual practices and unplanned pregnancy. Yet, countries have very different approaches to these services, and there are policy paradoxes even within country, such as abortion being free but contraception not so.

This analysis shows the importance of addressing European standards and criteria for children’s access to healthcare, and for ensuring a lack of economic or practical barriers. The Child Guarantee is not revolutionary—rather, Europe has hitherto failed to define actions or monitoring of agreed rights. As indicated in the Feasibility Study there is considerable scope to use European Commission tools and policy levers to facilitate moves in this direction and to support countries with problems or lack of expertise, but there is also a need within the healthcare sector to promote focussed research, knowledge sharing, and innovation, using mechanisms within the Statistical, Public Health, Research and DG Connect e-health fields.

This call for action is in line with the concern on children’s rights to health of the Lancet-UNICEF Commission on a Future for the World’s Children.^28^ Furthermore, in post-Covid-19 times, health system recovery will result in a major focus on strengthening adult services, meaning that children’s services will have to fight hard to keep their existing resources. To protect Europe’s children, it is vital for the EU and Member States to identify barriers to children’s healthcare provision, and to focus on actions to facilitate children’s access, and the initiatives necessary within healthcare and other sectors to achieve the Child Guarantee’s objective of free healthcare for all marginalized children of all ages.

*Conflicts of interest*: The author was Deputy PI of the MOCHA project, and health expert to the FSCG Consortium.

**Key points**

- The European Child Guarantee initiative proposes free healthcare for marginalized children, but without defining ‘free’ or ‘healthcare’.
- There is very little analysis of children’s healthcare costs and charges across the EU.
- Looking from birth to adolescence, only two EU-27 countries offered totally free healthcare, and two more address financial barriers.
- Disadvantaged families may also face other economic barriers to accessing healthcare, including travel and loss of earnings.
- Better data gathering, knowledge sharing and innovation in initiatives to address barriers to healthcare access by marginalized children, are needed.
